# Perceived barriers to seeking cancer care in the Gaza Strip: a cross-sectional study

**DOI:** 10.1186/s12913-020-06044-1

**Published:** 2021-01-06

**Authors:** Mohamedraed Elshami, Bettina Bottcher, Mohammed Alkhatib, Iyad Ismail, Khitam Abu-Nemer, Mustafa Hana, Ahmed Qandeel, Ahmed Abdelwahed, Hamza Yazji, Hisham Abuamro, Ghadeer Matar, Ahmed Alsahhar, Ahmed Abolamzi, Obay Baraka, Mahmood Elblbessy, Tahani Samra, Nabeela Alshorbassi, Alaa Elshami

**Affiliations:** 1 Harvard Medical School, Boston, MA USA; 2 Ministry of Health, Gaza, Palestine; 3 Faculty of Medicine, Islamic University of Gaza, Gaza, Palestine

**Keywords:** Cancer awareness, Barriers to cancer care, Early presentation, Patient survival, Low-resource setting, Low- and middle-income countries, Adolescents, Gaza, Palestine

## Abstract

**Background:**

Several factors might contribute to the delay to get cancer care including poor cancer awareness and having barriers to seeking help. This study described these barriers in Gaza and their association with recalling and recognizing cancer symptoms and risk factors.

**Methods:**

A cross-sectional study was conducted in Gaza. Adult visitors (≥18 years) to the largest three governmental hospitals and adolescent students (15–17 years) from ten high schools were recruited. A translated-into-Arabic version of the validated Cancer Awareness Measure (CAM) was used to collect data in face-to-face interviews. It described demographic data, barriers to seek cancer care as well as recall and recognition of cancer symptoms and risk factors. Responses were compared between adults and adolescents as well as males and females.

**Results:**

Of 3033 participants approached, 2886 completed the CAM (response rate= 95.2%). Among them, 1429 (49.5%) were adult (702 females; 49.1%) and 1457 (50.5%) were adolescent (781 females; 53.6%). The mean age± standard deviation (SD) of adult and adolescent participants was 33.7±11.7 years and 16.3±0.8 years, respectively. Emotional barriers were the most common barriers with ‘feeling scared’ as the most reported barrier (*n*=1512, 52.4%). Females and adolescents were more likely to report ‘feeling scared’ as a barrier than males and adults, respectively. Higher recall scores for cancer symptoms were associated with lower likelihood to report ‘embarrassment’, ‘worry about wasting doctor’s time’ and ‘difficulty arranging transport’. This was also seen for recalling risk factors, where ‘embarrassment’ and all practical barriers showed significant inverse associations with higher scores. In addition, greater recognition scores of cancer risk factors were inversely associated with reporting ‘embarrassment’ and ‘feeling scared’.

**Conclusions:**

The most commonly perceived barriers to seeking cancer care were ‘feeling scared’ and ‘feeling worried about what the doctor might find’, followed by practical and service barriers. Females and adolescents were more likely to report ‘fear’ as a barrier to seek medical advice. Having a higher recall of cancer symptoms and risk factors was inversely associated with reporting most barriers. To improve patient outcome, early presentation can be facilitated by targeting barriers specific to population groups.

**Supplementary Information:**

The online version contains supplementary material available at 10.1186/s12913-020-06044-1.

## Background

In the Gaza Strip, cancer accounts for 10.6% of the total reported deaths. It is the second leading cause of death after cardiovascular diseases [1]. The mortality rate of cancer increased from 24.0 to 36.1 per 100,000 general population between 2016 and 2018 [1].

Females represent 55.3% of cancer patients in the Gaza Strip with an age-adjusted rate of 169.0 per 100,000 female population, while males represent 44.7% with an age-adjusted rate of 162.0 per 100,000 male population [1, 2].

The cancer burden is expected to increase further in future, reaching levels that will severely impact the already limited financial and infrastructural resources of the healthcare system in Gaza [3]. In fact, cancer care that can be received in the governmental hospitals is restricted by many factors, including the availability of a small number of cancer specialists, limited access to medical technologies, and fluctuating funding from international organizations such as the World Health Organization and the United Nations Relief and Works Agency for Palestine Refugees (UNRWA) [3, 4].

Previous studies showed several factors contributing to the delay to get cancer care including poor awareness of cancer symptoms and risk factors and having different barriers that could be emotional, practical or service-related in nature [5, 6]. In addition to late presentation, inadequate access to healthcare services can result in more cancer-related deaths especially in low-resource settings as in Gaza [4, 7]. Therefore, the barriers to seeking cancer care of Palestinians living in Gaza must be explored in order to find effective approaches to mitigate their impact and enhance early presentation to health services and thus improving survival rates if cancer was diagnosed [8].

This study aimed to (i) identify people’s barriers to seeking cancer care in Gaza, (ii) compare the differences in the reported barriers between population groups, such as men and women as well as adults and adolescents, and (iii) test if there is an association between recalling/recognizing cancer symptoms and risk factors with having any of these perceived barriers.

## Methods

### Study design and population

This was a cross-sectional study conducted from the 1st of September to the 31st of October 2017 in the Gaza Strip, Palestine. It described people’s challenges and barriers to seek medical advice for cancer symptoms and tested the association of recalling and recognizing some cancer signs, symptoms, and risk factors with the reported barriers.

A translated-into-Arabic version of the Cancer Awareness Measure (CAM) questionnaire was used as an assessment tool, which was developed and validated for such purposes in the United Kingdom [9]. The questionnaire consisted of four sections; the first described the demographic data. The second reported the barriers to seeking cancer care. The barriers were further categorized into emotional, practical, and service barriers, and were compared between adolescents and adults as well as between males and females. The third section comprised two open-ended (recall) questions to list cancer signs/symptoms and risk factors while the fourth section included 16 closed (recognition) questions to identify eight cancer signs/symptoms and eight risk factors. A 3-point scale (no, I do not know, yes) was used to test the recognition of cancer signs/symptoms, while recognition questions of risk factors were based on a 5-point Likert scale (1= strongly disagree, 5= strongly agree).

Two bilingual experts translated the CAM from English to Arabic and another two bilingual experts back-translated it from Arabic to English. Before starting data collection, a pilot study was conducted with 119 respondents to test the clarity of the questions of the Arabic version of the CAM. Assessment of internal consistency was carried out on the used scale comprising 16 items. Cronbach’s alpha showed the questionnaire to reach acceptable reliability, α = 0.71.

### Sampling methods

Cancer care in the Gaza Strip is provided by governmental, non-governmental organizations, and a few private providers. Governmental hospitals are the main entry point for healthcare services for people who have medical insurance. The Palestinian government’s health insurance covers basic cancer therapies, while UNRWA sponsors the care of cancer patients by covering the cost of enrolling them in the government-funded health scheme [4]. Females represent 49.3% of the total population of the Gaza Strip, while adolescents represent about 23.0% [1]. There are 13 hospitals run by the Palestinian Ministry of Health in the Gaza Strip [1]. From these, the largest three hospitals, located in three geographical locations in the Gaza Strip, were selected to recruit participants by stratified sampling. Adults (≥ 18 years) admitted to or visiting those hospitals were the target population. Simple random sampling was used to recruit visitors or patients who were approached by data collectors on hospital grounds. Patients or visitors to the oncology departments at the participating hospitals were excluded from the study. A total of 1483 adult participants was approached in the included hospitals.

In parallel to this, stratified sampling was used to recruit adolescent participants from ten high schools (out of 147 high schools in Gaza [10]). Those high schools were in the same areas as the included hospitals to achieve uniformity of areas. Simple random sampling was then used to choose six classes in each high school. All students in the included 60 classes were invited to participate (*n*= 1550). Adolescents willing to participate were asked by data collectors to have a face-to face interview to fill out the CAM. The inclusion of both adults and adolescents gives the potential to have recommendations on future prevention interventions [5, 6].

According to the Palestinian Ministry of Health report in 2017 [11], there was a total of 190,208 adolescents in the Gaza Strip. Among those, 92,850 (48.8%) were females and 97,358 (51.2%) were males. In addition, there was a total of 901,640 adults, of whom 500,207 (55.5%) were women and 401,433 (44.5%) were men. Based on the sample size calculator of the Australian Bureau of Statistics [12], with a confidence level of 95.0% and a margin of error of 3.0%, the calculated minimum sample sizes were 1062 and 1066 for the strata of adolescents and adults, respectively. The calculated minimum sample sizes were 1066 and 1065 for strata of females and males, respectively. A total sample size of 2131 was needed.

Data collectors were trained to recruit participants and facilitate the completion of the CAM. They were also trained to clarify the questionnaire to illiterate participants. Prior to completing the questionnaire, a detailed explanation of the study including its purposes was given to the participants. Written informed consent was obtained from the participants and from their legal guardian if they were under 16 years old. Ethical approval had been obtained from both the Palestinian Ministry of Health and the Ministry of Education and Higher Education prior to data collection.

### Statistical analysis

Descriptive statistics were used for demographic data and the participants’ reported barriers. For every correctly recalled sign/symptom or risk factor, the participant was given one point. Moreover, participants who answered symptom recognition questions correctly (yes) were given one point and incorrect answers (no or do not know) were given no point. Similarly, people who responded to recognition questions by ‘agree’ or ‘strongly agree’ were given one point for every cancer risk factor. Those who answered with ‘disagree’, ‘strongly disagree’ or ‘not sure’ were given no point. The total for each of recall and recognition scores for cancer symptoms and risk factors was then calculated.

The chi-square test was used to compare barriers in males vs females and adults vs adolescents and to test the association between belonging to any of these population subgroups with having the reported barriers. Univariable logistic regression was utilized to have an idea about the magnitude of that association followed by a multivariable logistic regression to adjust for other covariates. The multivariable logistic regression was also used to test the association between recall/recognition scores for cancer symptoms and risk factors with having barriers to seeking cancer care. Results of the univariable and multivariable logistic regression were reported as odds ratios (OR) with corresponding 95% confidence intervals (CI) and *p*-values. Data were analyzed using Stata software version 16.0 (StataCorp, College Station, TX).

## Results

Of 3033 people who were invited to participate, 2886 participants completed the CAM questionnaire (response rate = 95.2%). Among them, 1457 (50.5%) were adolescent (781 females; 53.6%) and 1429 (49.5%) were adult (702 females; 49.1%). The adult mean age was 33.7 ± 11.7 and the adolescent mean age 16.3 ± 0.8 years (Table 1).

**Table 1:** Summary of characteristics of the participants

characteristic	n (%)	Mean age (±SD)
Gender		
Male	1403 (48.6)	24.9 (±11.5)
Female	1483 (51.4)	24.9(±12.2)
Age-group		
Adolescent	1457 (50.5)	16.3 (±0.8)
Adult	1429 (49.5)	33.7 (±11.4)
Total	2886 (100.0)	24.9 (±11.9)

*n* number of participants tested, *SD* standard deviation

### Barrier to seeking cancer care

Overall, emotional barriers were the most reported, followed by practical and service barriers (Table 2). Feeling scared was the most common barrier (*n*= 1512, 52.4%) followed by feeling worried about what a doctor might find (*n*= 1467, 50.8%). Females were more likely than males to feel scared and worried about the doctor’s findings (OR= 1.26; 95% CI: 1.09–1.47; *p*=0.002 and OR= 1.22; 95% CI: 1.04–1.42; *p*=0.013, respectively) after adjustment for age-group. Adolescents had more likelihood to report ‘feeling scared’ (OR= 1.28; 95% CI: 1.10–1.49; *p*=0.001) and ‘feeling insecure talking about symptoms with the doctor’ (OR= 1.64; 95% CI: 1.41–1.91; *p*< 0.001) than adults holding gender constant (Table 3). However, adolescents were less likely to feel worried about doctor’s findings than adults (OR= 0.57; 95% CI= 0.49–0.67; *p*=< 0.001).

**Table 2:** Summary of perceived barriers to seek cancer care in adolescents vs adults and females vs males

Type of Barrier	Barrier	Total (*n*= 2886) n (%)	Adolescents (*n*= 1457) n (%)	Adults (*n*= 1429) n (%)	***p***-value	Females (*n*= 1483) n (%)	Males (*n*= 1403) n (%)	***p***-value
Emotional	**Embarrassment**							
	Yes	1278 (44.3)	667 (45.8)	611 (42.8)	0.23	661 (44.6)	617 (44.0)	0.005
	No	1491 (51.7)	730 (50.1)	761 (53.2)		779 (52.5)	712 (50.7)	
	I do not know	117 (4.1)	60 (4.1)	57 (4.0)		43 (2.9)	74 (5.3)	
	**Feeling scared**							
	Yes	1512 (52.4)	800 (54.9)	712 (49.8)	< 0.001	827 (55.8)	685 (48.8)	< 0.001
	No	1241 (43.0)	576 (39.5)	665 (46.5)		603 (40.7)	638 (45.5)	
	I do not know	133 (4.6)	81 (5.6)	52 (3.6)		53 (3.5)	80 (5.7)	
	**Wouldn’t feel confident talking about symptom with doctor**							
	Yes	1402 (48.5)	774 (53.1)	628 (43.9)	< 0.001	706 (47.6)	696 (49.6)	0.07
	No	1250 (43.3)	539 (37.0)	711 (49.8)		669 (45.1)	581 (41.4)	
	I do not know	234 (8.1)	144 (9.9)	90 (6.3)		108 (7.3)	126 (9.0)	
	**Feeling worried about what a doctor might find**							
	Yes	1467 (50.8)	812 (55.7)	655 (45.8)	< 0.001	797 (53.7)	670 (47.8)	0.004
	No	1190 (41.3)	491 (33.7)	699 (48.9)		582 (39.3)	608 (43.3)	
	I do not know	229 (7.9)	154 (10.6)	75 (5.3)		104 (7.0)	125 (8.9)	
Service	**Feeling worried about wasting doctor’s time**				< 0.001			0.038
	Yes	581 (20.1)	276 (18.9)	305 (21.3)		280 (18.9)	301 (21.5)	
	No	2092 (72.5)	1039 (71.3)	1053 (73.7)		1105 (74.5)	987 (70.3)	
	I do not know	213 (7.4)	142 (9.8)	71 (5.0)		98 (6.6)	115 (8.2)	
	**Difficulty talking to doctor**							
	Yes	648 (22.5)	268 (18.4)	380 (26.6)	< 0.001	285 (19.2)	363 (25.9)	< 0.001
	No	1999 (69.3)	1032 (70.8)	967 (67.7)		1089 (73.4)	910 (64.9)	
	I do not know	239 (8.2)	157 (10.8)	82 (5.7)		109 (7.4)	130 (9.2)	
	**Difficulty making an appointment with the doctor**							
	Yes	900 (31.2)	484 (33.2)	416 (29.1)	< 0.001	445 (30.0)	455 (32.4)	< 0.001
	No	1767 (51.2)	827 (56.8)	940 (65.8)		949 (64.0)	818 (58.3)	
	I do not know	219 (7.6)	146 (10.0)	73 (5.1)		89 (6.0)	130 (9.3)	
Practical	**Busy to go to the doctor**							
	Yes	991 (34.3)	542 (37.2)	449 (31.4)	< 0.001	486 (32.8)	505 (36.0)	0.001
	No	1694 (58.7)	790 (54.2)	904 (63.3)		913 (61.6)	781 (55.7)	
	I do not know	201 (7.0)	125 (9.6)	76 (5.3)		84 (5.6)	117 (8.3)	
	**Having other things to do**							
	Yes	881 (30.5)	455 (31.2)	426 (29.8)	< 0.001	450 (30.3)	431 (30.7)	0.008
	No	1774 (61.5)	850 (58.3)	924 (64.7)		936 (63.1)	838 (59.7)	
	I do not know	231 (8.0)	152 (10.5)	79 (5.5)		97 (6.6)	134 (9.6)	
	**Difficulty arranging transports**							
	Yes	979 (33.9)	466 (32.0)	513 (35.9)	< 0.001	535 (36.1)	444 (31.6)	0.002
	No	1697 (58.8)	853 (58.5)	844 (59.1)		861 (58.1)	836 (59.6)	
	I do not know	210 (7.3)	138 (9.5)	72 (5.0)		87 (5.8)	123 (8.8)	

*n* number of participants tested

**Table 3:** The association of being a female or an adolescent with reporting barriers to seek medical advice for cancer

Type of barrier	Barrier	Female gender				Being adolescent			
		Unadjusted OR (95% CI)	***p***-value	^a^Adjusted OR (95% CI)	***p***-value	Unadjusted OR (95% CI)	***p***-value	^b^Adjusted OR (95% CI)	***p***-value
Emotional	**Embarrassment (*n*= 2769)**	0.98 (0.84–1.14)	0.78	0.97 (0.84–1.13)	0.72	1.14 (0.98–1.32)	0.09	1.14 (0.98–1.32)	0.09
	**Feeling scared (*n*= 2753)**	1.28 (1.10–1.48)	**0.001**	1.26 (1.09–1.47)	**0.002**	1.30 (1.12–1.51)	**0.001**	1.28 (1.10–1.49)	**0.001**
	**Wouldn’t feel confident talking about symptom with doctor (*n*= 2652)**	0.88 (0.76–1.03)	0.10	0.86 (0.73–1.01)	0.051	1.63 (1.39–1.90)	**< 0.001**	1.64 (1.41–1.91)	**< 0.001**
	**Feeling worried about what a doctor might find (*n*= 2657)**	1.24 (1.07–1.45)	**0.005**	1.22 (1.04–1.42)	**0.013**	0.57 (0.49–0.66)	**< 0.001**	0.57 (0.49–0.67)	**< 0.001**
Service	**Feeling worried about wasting doctor’s time (*n*= 2673)**	0.83 (0.69–0.99)	**0.048**	0.83 (0.69–1.01)	0.052	1.09 (0.91–1.31)	0.36	1.08 (0.90–1.30)	0.40
	**Difficulty talking to doctor (*n*= 2647)**	0.66 (0.55–0.78)	**< 0.001**	0.67 (0.56–0.80)	**< 0.001**	1.51 (1.26–1.81)	**< 0.001**	1.48 (1.24–1.78)	**< 0.001**
	**Difficulty making an appointment with the doctor (*n*= 2667)**	0.84 (0.72–0.99)	**0.037**	0.83 (0.71–0.97)	**0.023**	0.76 (0.64–0.89)	**0.001**	0.75 (0.64–0.88)	**< 0.001**
Practical	**Busy to go to the doctor (*n*= 2685)**	0.82 (0.70–0.96)	**0.015**	0.81 (0.69–0.94)	**0.008**	1.38 (1.18–1.62)	**< 0.001**	1.40 (1.19–1.64)	**< 0.001**
	**Having other things to do (*n*= 2655)**	0.93 (0.80–1.10)	0.41	0.93 (0.79–1.09)	0.36	1.16 (0.99–1.36)	0.07	1.17 (0.99–1.37)	0.06
	**Difficulty arranging transports to the doctor (*n*= 2676)**	1.17 (0.99–1.37)	0.051	1.18 (1.01–1.38)	**0.042**	1.11 (0.95–1.30)	0.18	1.12 (0.96–1.32)	0.15

*n* number of participants tested, *OR* odds ratio, *CI* confidence interval

Notes

^a^Adjusted for age group

^b^Adjusted for gender

To overcome the non-linear relationships, the ‘I do not know’ category of each barrier was dropped for easier interpretation

*p*-values in bold are less than 0.05

Difficulty making an appointment with the doctor was the most frequently reported service barrier (*n*=900, 31.2%). In the multivariable logistic regression, both females and adolescents were less likely than males and adults to report difficulty making an appointment with the doctor as a barrier to seek medical advice (OR= 0.83; 95% CI: 0.71–0.97; *p*=0.023 and OR= 0.75; 95% CI: 0.64–0.88; *p*< 0.001, respectively). However, men and adolescents showed more likelihood than women and adults to find difficulty talking to the doctor (OR=0.67; 95% CI: 0.56–0.80; p< 0.001 and OR= 1.48; 95% CI: 1.24–1.78; p< 0.001, respectively). Being busy to go to the doctor was the most observed practical barrier (*n*=991, 34.3%) followed by difficulty arranging transport (*n*=979, 33.9%). Females had a lower likelihood than males to be busy to go to the doctor (OR= 0.81; 95% CI: 0.69–0.94; *p*=0.008), while adolescents were more likely than adults to have this barrier (OR= 1.40; 95% CI: 1.19–1.64; *p*< 0.001).

### Relationship between recalling/recognizing cancer symptoms and reporting barriers to seek cancer care

After adjusting for age and gender, participants who had higher recall scores for cancer symptoms were found to have a decrease in the likelihood to report ‘embarrassment’ (OR= 0.90, 95% CI: 0.82–0.97, *p*=0.010), ‘feeling worried about doctor’s findings’ (OR= 0.89, 95% CI: 0.80–0.98, *p*=0.024) and ‘difficulty arranging transport’ (OR= 0.91, 95% CI: 0.83–0.99, *p*=0.034) than those with lower scores (Table 4). No associations were noticed between recognition scores and reporting any barrier.

**Table 4:** The association of overall recall/recognition scores for cancer symptoms with reporting barriers to seek medical advice for cancer

Type of barrier	Barrier	Overall recall score				Overall recognition score			
		Unadjusted OR (95% CI)	***p***-value	^a^Adjusted OR (95% CI)	***p***-value	Unadjusted OR (95% CI)	***p***-value	^a^Adjusted OR (95% CI)	***p***-value
Emotional	**Embarrassment (*n*= 2769)**	0.89 (0.82–0.97)	**0.008**	0.90 (0.82–0.97)	**0.010**	1.01 (0.97–1.06)	0.53	1.02 (0.98–1.06)	0.39
	**Feeling scared (*n*= 2753)**	1.06 (0.98–1.15)	0.16	1.05 (0.97–1.14)	0.26	0.97 (0.93–1.01)	0.19	0.98 (0.93–1.02)	0.26
	**Wouldn’t feel confident talking about symptom with doctor (*n*= 2652)**	0.98 (0.90–1.07)	0.71	0.99 (0.92–1.09)	0.99	0.96 (0.92–1.00)	0.08	0.98 (0.94–1.02)	0.37
	**Feeling worried about what a doctor might find (*n*= 2657)**	1.08 (0.99–1.17)	0.08	1.07 (0.98–1.17)	0.12	0.98 (0.94–1.03)	0.42	0.99 (0.95–1.04)	0.90
Service	**Feeling worried about wasting doctor’s time (*n*= 2673)**	0.88 (0.79–0.97)	**0.013**	0.89 (0.80–0.98)	**0.024**	0.97 (0.92–1.03)	0.31	0.98 (0.93–1.03)	0.36
	**Difficulty talking to doctor (*n*= 2647)**	0.90 (0.81–0.99)	**0.03**	0.91 (0.82–1.01)	0.07	1.01 (0.96–1.06)	0.79	0.99 (0.95–1.05)	0.93
	**Difficulty making an appointment with the doctor (*n*= 2667)**	0.90 (0.83–0.99)	**0.027**	0.92 (0.84–1.01)	0.07	1.02 (0.98–1.07)	0.38	1.04 (0.99–1.09)	0.14
Practical	**Busy to go to the doctor (*n*= 2685)**	0.92 (0.84–1.00)	0.06	0.93 (0.86–1.02)	0.13	1.01 (0.97–1.06)	0.62	1.03 (0.98–1.08)	0.23
	**Having other things to do (*n*= 2655)**	0.93 (0.85–1.02)	0.12	0.94 (0.86–1.02)	0.15	1.03 (0.98–1.08)	0.22	1.04 (0.99–1.08)	0.14
	**Difficulty arranging transports to the doctor (*n*= 2676)**	0.92 (0.84–1.00)	0.06	0.91 (0.83–0.99)	**0.034**	1.02 (0.98–1.07)	0.31	1.02 (0.98–1.07)	0.35

*n* number of participants tested, *OR* odds ratio, *CI* confidence interval

Notes

^a^Adjusted for age and gender.

To overcome the non-linear relationships, the ‘I do not know’ category of each barrier was dropped for easier interpretation

*p*-values in bold are less than 0.05

### Relationship between recalling/recognizing cancer risk factors and reporting barriers to seek cancer care

People who had greater recall scores for cancer risk factors were less likely to report ‘embarrassment’ (OR= 0.89, 95% CI: 0.82–0.89, *p*=0.013), ‘being busy to go to the doctor’ (OR= 0.88, 95% CI: 0.80–0.97, *p*=0.008), ‘having other things to do’ (OR= 0.87, 95% CI: 0.79–0.96, *p*=0.005) and ‘difficulty arranging transport’ (OR= 0.83, 95% CI: 0.76–0.92, *p*< 0.001) than those with lower scores after adjustment to age and gender (Table 5). In addition, participants who had higher recognition scores had a lower likelihood to report ‘embarrassment’ (OR= 0.95, 95% CI: 0.91–0.99, *p*=0.035) and ‘feeling scared’ (OR= 0.88, 95% CI: 0.80–0.97, *p*=0.010) than participants with lower scores holding age and gender constant.

**Table 5:** The association of overall recall/recognition scores for cancer risk factors with reporting barriers to seek medical advice for cancer

Type of barrier	Barrier	Overall recall score				Overall recognition score			
		Unadjusted OR (95% CI)	***p***-value	^a^Adjusted OR (95% CI)	***p***-value	Unadjusted OR (95% CI)	***p***-value	^a^Adjusted OR (95% CI)	***p***-value
Emotional	**Embarrassment (*n*= 2769)**	0.89 (0.82–0.98)	**0.015**	0.89 (0.82–0.89)	**0.013**	0.95 (0.90–0.99)	**0.016**	0.95 (0.91–0.99)	**0.035**
	**Feeling scared (*n*= 2753)**	0.98 (0.90–1.07)	0.64	0.97 (0.89–1.06)	0.51	0.94 (0.90–0.98)	**0.005**	0.94 (0.90–0.99)	**0.010**
	**Wouldn’t feel confident talking about symptom with doctor (*n*= 2652)**	0.93 (0.85–1.02)	0.14	0.93 (0.85–1.02)	0.12	0.96 (0.91–1.00)	0.07	0.99 (0.94–1.03)	0.56
	**Feeling worried about what a doctor might find (*n*= 2657)**	0.98 (0.90–1.07)	0.68	0.97 (0.88–1.06)	0.47	0.97 (0.93–1.02)	0.24	0.99 (0.95–1.05)	0.91
Service	**Feeling worried about wasting doctor’s time (*n*= 2673)**	0.92 (0.83–1.03)	0.15	0.93 (0.83–1.04)	0.18	0.98 (0.93–1.04)	0.49	0.98 (0.94–1.03)	0.59
	**Difficulty talking to doctor (*n*= 2647)**	0.95 (0.85–1.05)	0.31	0.96 (0.86–1.07)	0.46	0.99 (0.95–1.05)	0.91	0.98 (0.93–1.04)	0.49
	**Difficulty making an appointment with the doctor (*n*= 2667)**	0.91 (0.83–1.00)	0.06	0.91 (0.83–1.00)	0.06	1.02 (0.97–1.07)	0.37	1.05 (0.99–1.10)	0.07
Practical	**Busy to go to the doctor (*n*= 2685)**	0.88 (0.80–0.97)	**0.009**	0.88 (0.80–0.97)	**0.008**	0.97 (0.92–1.01)	0.16	0.99 (0.94–1.04)	0.64
	**Having other things to do (*n*= 2655)**	0.87 (0.79–0.96)	**0.005**	0.87 (0.79–0.96)	**0.005**	0.96 (0.92–1.01)	0.12	0.97 (0.92–1.02)	0.22
	**Difficulty arranging transports to the doctor (*n*= 2676)**	0.84 (0.76–0.92)	**< 0.001**	0.83 (0.76–0.92)	**< 0.001**	0.99 (0.94–1.04)	0.68	0.99 (0.94–1.03)	0.56

*n* number of participants tested, *OR* odds ratio, *CI* confidence interval

Notes

^a^Adjusted for age and gender

To overcome the non-linear relationships, the ‘I do not know’ category of each barrier was dropped for easier interpretation

p-values in bold are less than 0.05

## Discussion

‘Feeling scared’ and ‘feeling worried about what the doctor might find’ were the most common perceived barriers among Palestinians living in the Gaza Strip. Females and adolescents were more likely to report ‘feeling scared’ as a barrier than males and adults, respectively. On the contrary, females and adolescents were less likely than males and adults to report difficulty making an appointment. Adolescents were more likely to report ‘busy to go to the doctor’ as a practical barrier to seek medical advice. Significant associations between recall scores of cancer symptoms and reported barriers to early presentation were found for ‘embarrassment’, ‘worry about doctor’s findings’ and ‘difficulty arranging transports’, where higher recall scores were associated with lower likelihood to report each barrier. On the other hand, recognition scores for cancer symptoms showed no significant associations. Reporting ‘embarrassment’ and all practical barriers showed significant associations, where higher recall of cancer risk factors was associated with less likelihood to have these barriers.

### Impact of knowledge on perceived barriers to seek medical advice for cancer

Delay to cancer diagnosis can occur in any or all of three stages; presentation to the doctor (also called the patient interval), diagnosis, or access to treatment [13]. While diagnosis and treatment intervals were found to be comparable in low- and middle-income countries (LMICs) with those in high-income countries, the patient interval was found to be longer in LMICs [14, 15]. A prolonged patient interval is a significant contributor to worse patient outcomes and a factor for 70% of global cancer mortality occurring in LMICs [16, 17]. Poorer knowledge of cancer signs and symptoms was found to prolong the patient interval in LMICs as well as high-income countries [5, 6, 18–28]. This might be at least partly caused by the fact that less knowledge may drive people to have more barriers to early presentation and vice versa. In this study, too, participants with greater knowledge of cancer symptoms were less likely to report barriers, especially ‘embarrassment’ and ‘feeling worried about doctor’s findings’, illustrating the empowerment knowledge can give to people when seeking care for possible cancer symptoms. Furthermore, participants with better awareness of cancer risk factors also reported significantly less service barriers, demonstrating that greater knowledge of cancer risk factors has the potential to reduce barriers to seeking medical advice and, thus, promote early presentation.

### ‘Fear’ and ‘worry’ – related barriers

Prevalent negative beliefs among the population around a cancer diagnosis could be one reason for the greater impact of ‘fear’ and ‘worry’ on reported barriers in LMICs compared to high-income countries, where practical barriers were most frequently reported [17, 20, 29–31]. As in other low-income settings, stronger negative beliefs could be associated with a cancer diagnosis and could result from experiences of higher mortality and less chances of cure [32–36]. This assumption is underpinned by the fact that the most frequently reported barrier was ‘feeling scared’, illustrating the belief of cancer to be fatal [37–39]. This is difficult as late presentation prolongs the patient interval and, hence, also the time to diagnosis, exerting further negative impact on patient outcomes and reducing chances of cure [14, 17, 40]. In fact, in the Gaza Strip, 60% of the deaths due to breast cancer in 2016 (643 of 1072 deaths) were found to be avoidable, often due to late diagnosis [41], underlining the justified basis for negative beliefs to rise and disseminate within the population. Therefore, barriers can be reinforced by local experience and beliefs [42–44]. On the other hand, improved knowledge on cancer treatments and outcomes could address negative beliefs and remove barriers to presentation and by this promote early presentation. Participants with more knowledge about cancer risk factors reported less practical barriers to seek medical advice, such as ‘difficulty arranging transport to see the doctor’ or ‘having other things to do’, highlighting the fact that increased knowledge may play a role in mitigating barriers. Increased public knowledge of cancer symptoms, risk factors as well as management could be achieved by a combination of public awareness campaigns and individual interventions on school and community levels, which has been shown to be effective in high-income countries [45–49]. Funds are usually lacking for such interventions in LMICs; however, in the long-term, reduced morbidity and mortality might make such investments cost-effective also for low-income settings [50–52].

### Differences and similarities related to gender and age

Women and adolescents were more likely to report ‘fear’ as a barrier than men and adults, which has also been found by other studies [27, 53]. Adolescents were also found in other studies to express ‘fear’ as an important barrier [54–56]. A school-based intervention program, aimed at increasing knowledge on cancer signs and symptoms was able to reduce such barriers, at least in the short-term [48]. Although adolescents were shown to retain the knowledge after six months, this knowledge had lost its positive effect on reducing barriers after that time. Therefore, effective intervention programs can work for adults and adolescents but must address knowledge as well as barriers to seeking help for greatest impact.

In this study, adolescents reported difficulty talking to the doctor, possibly due to a disinclination to express emotions to healthcare professionals or inexperience in doing so [57]. Men and adults identified difficulties in making an appointment with the doctor significantly more than women and adolescents. Women engage generally more with the healthcare system in Gaza than men, as women take up medical care regularly during pregnancies and childbirths and, thus, have more experience in making appointments with doctors. These differences illustrate that next to knowledge also culture influences health-seeking behaviour. Therefore, while good knowledge is associated with having less perceived barriers to help-seeking with cancer symptoms, it is not the only factor. In order to significantly reduce the patient interval, barriers to seeking medical advice have to also be addressed to promote early diagnosis and reduce excess mortality from cancer [17, 41].

### Future directions

In order to improve survival outcomes for cancer in low-income settings, like the Gaza Strip, it is essential to reduce the interval to diagnosis by promoting early presentation with symptoms. Essential interventions to achieve this have to focus on reducing barriers to presentation, such as ‘embarrassment’ or ‘fear’ or ‘worry about what the doctor might find’. This can be achieved by nurturing greater public awareness and including more health content in school curricula, as greater knowledge was associated with less barriers in this study. Furthermore, reducing practical barriers by improving access to healthcare as well as the quality of care available to people will reduce barriers of ‘fear’ and ‘difficulty making an appointment with a doctor’, which were noticed in this study.

### Strengths and limitations

This study had a large sample size and could be considered representative of the Gaza population. The study also had a high response rate and used a validated instrument, the CAM. In addition, the inclusion of both adults and adolescents provides the opportunity for additional recommendations on prevention interventions.

However, adult participants were mainly recruited from among the visitors to governmental hospitals (excluding oncology departments). This might have led to recruitment of people displaying a certain degree of help-seeking behaviour and, thus, leading to some degree of selection bias. On the other hand, hospitals are busy places in the Gaza Strip with two, three or more visitors present with patients at all times, in addition to relatives waiting outside for news. Therefore, visitors did not necessarily display help-seeking behaviour themselves. Furthermore, this study surveyed healthy participants, who had not experienced a cancer diagnosis and, hence, looked at ‘anticipated’ feelings and behaviours. This could differ from actual feelings, experiences and behaviours in presence of cancer symptoms. In order to look at this more closely, further research should examine potential delay in the three stages of presentation, diagnosis and treatment experienced by cancer patients.

## Conclusion

The most commonly reported barriers to seeking cancer care were ‘feeling scared’ and ‘feeling worried about what the doctor might find’, followed by practical and service barriers. In this study, females and adolescents were more likely to report ‘fear’ as a barrier to seek medical advice. Having a higher recall of cancer symptoms and risk factors was inversely associated with reporting most barriers to seek cancer care. In order to improve cancer outcomes in LMICs, such as the Gaza Strip, reducing the patient interval is essential. This can be achieved by targeting interventions to population groups with view to their specific barriers.

## Supplementary Information

**Additional file 1.**

## Data Availability

The dataset used and analyzed during the current study is available from the corresponding author on reasonable request.

## Abbreviations

UNRWA: United Nations Relief and Works Agency
CAM: cancer awareness measure
LMICs: low- and middle-income countries
CI: confidence interval
OR: Odds ratio
