# Supplementary material for: Perceived barriers to seeking cancer care in the Gaza Strip: a cross-sectional study
Source: BMC Health Serv Res. 2021 Jan 6;21:28. doi: 10.1186/s12913-020-06044-1 (PMC7788935; doi:10.1186/s12913-020-06044-1)
Supplement: Supplementary file 1 — Additional file 1. [file 12913_2020_6044_MOESM1_ESM.docx]

**Cancer Awareness Questionnaire**

Serial number: ……………….

Data collection site: ……………….

**Section (1): demographic data**

Age: …………. Years

Gender: 🞏 Female 🞏 Male

**Section (2): perceived barriers to seeking cancer care**

| Sometimes people put off going to see the doctor, even when they have a symptom suggestive of cancer. Could you say if any of these might put you off going to the doctor? | | | |
| --- | --- | --- | --- |
|  | Yes | No | Do not know |
| 1. You would be too embarrassed |  |  |  |
| 1. You would be too scared |  |  |  |
| 1. You would be worried about wasting the doctor’s time |  |  |  |
| 1. Your doctor would be difficult to talk to |  |  |  |
| 1. It would be difficult to make an appointment with your doctor |  |  |  |
| 1. You would be too busy to make time to go to the doctor |  |  |  |
| 1. You have too many other things to worry about |  |  |  |
| 1. It would be difficult for you to arrange transport to the doctor’s place |  |  |  |
| 1. You would be worried about what the doctor might find |  |  |  |
| 1. You would not feel confident talking about your symptom with the doctor |  |  |  |

**Section (3): recall of cancer signs, symptoms, and risk factors**

- There are many warning signs and symptoms of cancer. Please name as many as you can think of:
- What things do you think affect a person’s chance of developing cancer?

**Section (4): recognition of cancer signs, symptoms, and risk factors**

| The following may or may not be warning signs for cancer. We are interested in your opinion: | | | |
| --- | --- | --- | --- |
|  | **Yes** | **No** | **Do not know** |
| 1. Do you think an unexplained lump or swelling could be a sign of cancer? |  |  |  |
| 1. Do you think persistent unexplained pain could be a sign of cancer? |  |  |  |
| 1. Do you think unexplained bleeding could be a sign of cancer? |  |  |  |
| 1. Do you think a persistent cough or hoarseness could be a sign of cancer? |  |  |  |
| 1. Do you think a persistent change in bowel or bladder habits could be a sign of cancer? |  |  |  |
| 1. Do you think persistent difficulty swallowing could be a sign of cancer? |  |  |  |
| 1. Do you think a sore that does not heal could be a sign of cancer? |  |  |  |
| 1. Do you think unexplained weight loss could be a sign of cancer? |  |  |  |

| These are some of the things that can increase a person’s chance of developing cancer. How much do you agree that each of these can increase a person’s chance of developing cancer? | | | | | |
| --- | --- | --- | --- | --- | --- |
|  | **1= Strongly disagree** | **2= Disagree** | **3=**  **Not sure** | **4= Agree** | **5= Strongly agree** |
| 1. Smoking any cigarettes at all |  |  |  |  |  |
| 1. Exposure to another person’s cigarette smoke |  |  |  |  |  |
| 1. Eating less than 5 portions of fruit and vegetables a day |  |  |  |  |  |
| 1. Being overweight |  |  |  |  |  |
| 1. Getting sunburnt more than once as a child |  |  |  |  |  |
| 1. Being over 70 years old |  |  |  |  |  |
| 1. Having a close relative with cancer |  |  |  |  |  |
| 1. Doing less than 30 mins of moderate physical activity 5 times a week |  |  |  |  |  |
